# Особенности взаимосвязи параметров микробиоты кишечника с клиническими и биохимическими показателями у лиц с ожирением молодого возраста

**DOI:** 10.14341/probl13454

**Published:** 2024-09-16

**Authors:** Т. С. Душина, Л. А. Суплотова, С. М. Кляшев, М. В. Николенко, Е. Ф. Дороднева

**Affiliations:** Тюменский государственный медицинский университет; Тюменский государственный медицинский университет; Тюменский государственный медицинский университет; Тюменский государственный медицинский университет; Тюменский государственный медицинский университет

**Keywords:** микробиота кишечника, ожирение, колонофлор-16 (премиум), Faecalibacterium prausnitzii, Fusobacterium nucleatum, Prevotella spp

## Abstract

**АКТУАЛЬНОСТЬ:**

АКТУАЛЬНОСТЬ. Заболеваемость ожирением драматически растет во всем мире. В последнее время появляется все больше доказательств связи ожирения с функциональным состоянием микробиоты кишечника. Понимание этой взаимосвязи может обеспечить новые подходы к лечению ожирения путем управления качественными и количественными параметрами бактериально-грибковых ассоциаций кишечника.

**ЦЕЛЬ:**

ЦЕЛЬ. Изучить особенности качественного и количественного состава микробиоты толстого кишечника и оценить ассоциации с анамнестическими, антропометрическими и биохимическими показателями у молодых пациентов с ожирением.

**МАТЕРИАЛЫ И МЕТОДЫ:**

МАТЕРИАЛЫ И МЕТОДЫ. Проведено одноцентровое поперечное одномоментное контролируемое исследование с участием 118 молодых людей, из которых 87 человек имели ожирение, и 31 человек с нормальной массой тела составлял группу контроля. Всем участникам проводился биохимический анализ крови (общий холестерин, липопротеиды высокой плотности, липопротеиды низкой плотности, липопротеиды очень низкой плотности, триглицериды, мочевая кислота, глюкоза, гликированный гемоглобин, С-реактивный белок), а также оценка состояния микробиоты толстой кишки методом полимеразной цепной реакции в режиме реального времени с применением набора реактивов «Колонофлор-16 (премиум)». Для статистических расчетов был использован пакет прикладных программ Microsoft Excel 2010, IBM SPSS Statistics 26.0. Результаты оценивались как статистически значимые при уровне р<0,05.

**РЕЗУЛЬТАТЫ:**

РЕЗУЛЬТАТЫ. Анализируя результат «Колонофлор-16 премиум», выявлено расхождение полученных данных группы контроля с референсными значениями анализа. В группе пациентов с ожирением наблюдалась отчетливая тенденция к снижению содержания Lactobacillus spp и Bifidobacterium spp. Кроме того, в сравнении с группой контроля (10,3%) в группе ожирения достоверно превалирует Fusobacterium nucleatum (37,6%) (p=0,005) при достоверном снижении бактерий Faecalibacterium prausnitzii (p=0,030) и повышении бактерий Prevotella spp (p=0,029). У молодых пациентов с ожирением выявлены многочисленные ассоциации представителей микробиоты толстой кишки с важнейшими анамнестическими, антропометрическими и биохимическими параметрами.

**ЗАКЛЮЧЕНИЕ:**

ЗАКЛЮЧЕНИЕ. Наблюдается перераспределение филотипов микробиоты, характеризующееся уменьшением апатогенных микроорганизмов и появлением и увеличением условно-патогенных и патогенных микроорганизмов, что в целом свидетельствует о формировании провоспалительного потенциала доминантов и ассоциантов у молодых пациентов с ожирением. Наличие статистически значимых корреляционных зависимостей убедительно свидетельствует о существовании тесных и многообразных взаимосвязей количественных и качественных параметров микробиоты с метаболическими параметрами пациентов.

## Abstract (en)

**BACKGROUND:**

BACKGROUND: The prevalence of obesity is rising dramatically worldwide. Recently, there is increasing evidence linking obesity with the functional state of the intestinal microbiota. The understanding of this relationship may provide new approaches to the treatment of obesity by manipulating the qualitative and quantitative parameters of intestinal bacterial-fungal associations.

**AIM:**

AIM: To study the features of the qualitative and quantitative composition of the colon microbiota and to evaluate associations with anamnestic, anthropometric and biochemical parameters in young obese patients.

**MATERIALS AND METHODS:**

MATERIALS AND METHODS: A single-center, cross-sectional, single-stage, controlled study was conducted with the participation of 118 young people, of whom 87 were obese, and 31 people with normal body weight formed the control group. All participants underwent a biochemical blood test (total cholesterol, high-density lipoproteins, low-density lipoproteins, very low-density lipoproteins, triglycerides, uric acid, glucose, glycated hemoglobin, C-reactive protein), as well as an assessment of the state of the colon microbiota using polymerase chain reaction in real time using a set of Colonoflor-16 (premium) reagents. The Microsoft Excel 2010 and IBM SPSS Statistics 26.0 application software package was used for statistical calculations. The results were evaluated as statistically significant at a level of p<0.05.

**RESULTS:**

RESULTS: Analyzing the result of Colonoflor-16 premium, the discrepancy between the obtained data of the control group and the reference values of the analysis was revealed. There was a clear tendency to decrease the content of Lactobacillus spp and Bifidobacterium spp in the obesity group. In addition, in comparison with the control group (10.3%), in the obesity group Fusobacterium nucleatum significantly prevailed (37.6%) (p=0.005), with a significant decrease in the bacteria Faecalibacterium prausnitzii (p=0.030), and an increase in the bacteria Prevotella spp (p=0.029). A lot of associations of representatives of the colon microbiota with the most important anamnestic, anthropometric and biochemical parameters were revealed in young obese patients.

**CONCLUSION:**

CONCLUSION: There is a redistribution of microbiota phylotypes characterized by a decrease in apathogenic microorganisms and the appearance and increase of opportunistic and pathogenic microorganisms, which generally indicates the formation of the pro-inflammatory potential of dominants and associates in young obese patients. The presence of statistically significant correlations strongly indicates of existence of close and diverse relationships between the quantitative and qualitative parameters of the microbiota and the metabolic parameters of patients.

## АКТУАЛЬНОСТЬ

В последние десятилетия заболеваемость ожирением продолжает расти с угрожающей скоростью. Почти два миллиарда взрослых во всем мире имеют избыточный вес, более половины из них страдают ожирением [1].

Нарастание глобальной эпидемии ожирения связано с увеличением заболеваемости сахарным диабетом 2 типа (СД2), неалкогольной жировой болезнью печени (НАЖБП) и некоторыми видами рака, что во многом обусловлено ростом инсулинорезистентности при данной патологии [2]. Следовательно, ожирение является одной из основных причин заболеваемости, смертности и расходов на здравоохранение, что заставляет искать новые подходы к изучению его патогенетических механизмов с целью разработки новых таргетных подходов к профилактике и лечению.

За последние несколько десятилетий резко возросло количество исследований, изучающих роль микробиоты кишечника в развитии ожирения и связанных с ним метаболических заболеваний. Микробиоту кишечника можно охарактеризовать как удивительный «новый орган», который состоит примерно из 100 триллионов организмов, включая бактерии, вирусы, грибы и бактериофаги [3]. Составляя примерно 1 кг от общей массы тела, микробиота кишечника регулирует важные метаболические процессы в организме, к которым можно отнести усвоение питательных веществ и регуляцию использования энергии. Помимо этого, ассоциативные микроорганизмы поддерживают целостность кишечного барьера (слизистой оболочки кишечника) за счет синтеза муцина и регенерации эпителия, стимулируют секреторный иммунный ответ, регулируют биодеградацию полисахаридов, участвуют в синтезе витаминов и незаменимых аминокислот, опосредуют выработку короткоцепочечных жирных кислот (КЦЖК) [4].

Первые доказательства связи между качественным и количественным составом микробиоты кишечника и ожирением были получены в исследованиях на стерильных мышах. Взрослым стерильным мышам GF (англ. Germ-free) трансплантировали кишечную микробиоту, полученную из слепой кишки мышей с ожирением, выращенных традиционным способом. В течение 14 дней у GF-мышей общее содержание жира в организме увеличилось на 60%, и развилась инсулинорезистентость несмотря на то, что они потребляли меньшее количество пищи [5]. При предоставлении, как GF-особям, так и обычным мышам, доступа к неограниченному питанию было замечено, что GF-мыши демонстрировали значительно меньшее (42%) накопление общего жира в организме, чем обычные мыши, несмотря на то, что ежедневно потребляли на 29% больше еды [5]. Безмикробные мыши (GF) демонстрировали свойства, указывающие на возможность устойчивости к развитию ожирения, вызванного употреблением высококалорийной диеты, что убедительно указывало на возможную причинную роль кишечной микробиоты.

Ридаура и др. [6] были первой группой исследователей, которые выполнили трансплантацию фекальной микробиоты человека на когорте GF-грызунов. Кишечную микробиоту получали от взрослых женщин-близнецов, дискордантных по ожирению. Первоначально все GF-грызуны имели нормальный вес, получали нежирный корм и содержались совместно. Грызуны, которым была трансплантирована микробиота от тучных пациентов, продемонстрировали рост общей массы тела и жировой массы, в то время как те, кому трансплантировали микробиоту от здоровых людей, остались худыми. Секвенирование стула грызунов показало успешную интеграцию донорской микробиоты человека [6].

Что касается специфического микробного состава у пациентов с ожирением и без, то результаты исследований демонстрируют противоречивые данные. Некоторые исследования, сравнивающие людей с ожирением и худощавых, показали более высокую численность бактерий из отдела Firmicutes (Blautia spp., Hydrogenotorophica spp., Coprococcus catus, Eubacterium ventriosum, Ruminococcus bromii и Ruminococcus obeum) у людей с ожирением, тогда как у людей с нормальным весом было больше бактерий из Bacteroidetes spp. (Bacteroides faecichinchillae и Bacteroides thetaiotaomicron), что приводило к более высокому соотношению Firmicutes/Bacteroidetes [7]. В то же время в других исследованиях сообщается об уменьшении соотношения Firmicutes/Bacteroidetes у людей с ожирением [8].

Ряд исследований продемонстрировали, что ожирение ассоциировано со снижением микробного богатства и разнообразия микробиоты кишечника [9][10]. Измененная микробиота кишечника, предположительно, участвует в патогенезе ожирения посредством множества механизмов, включая нарушение энергетического гомеостаза, синтеза и хранения липидов, центральной регуляции аппетита и пищевого поведения, а также хронического низкоуровневого воспаления [11][12].

Таким образом, есть представление о существовании уменьшения разнообразия кишечной микробиоты у лиц с ожирением, однако до сих пор нет полных объективных данных о точной микробной популяции микробиоты кишечника при данной патологии. Понимание взаимосвязи между микробиотой кишечника и потенциальными механизмами развития ожирения может обеспечить новые подходы к лечению.

## ЦЕЛЬ

Изучить особенности качественного и количественного состава микробиоты толстого кишечника и оценить ассоциации с анамнестическими, антропометрическими и биохимическими показателями у молодых пациентов с ожирением.

## МАТЕРИАЛЫ И МЕТОДЫ

## Место и время проведения исследования

Место проведения. Университетская многопрофильная клиника ФГБОУ ВО «Тюменский государственный медицинский университет» Министерства здравоохранения Российской Федерации, Тюмень, Россия.

Время проведения. Апрель–сентябрь 2023 г.

## Изучаемые популяции

Изучалось две популяции: лица с ожирением (индекс массы тела (ИМТ) — 37,2 [ 34,1; 42,05] кг/м²) и лица с нормальной массой тела (ИМТ — 21,9 [ 20,2; 23,5] кг/м²).

Критерии включения для больных с ожирением: возраст от 18 до 44 лет, подписание информированного согласия, стабильная масса тела (изменение менее 10% массы тела за 3 месяца до исследования), отсутствие соматической патологии.

Критерии включения для лиц группы контроля: возраст от 18 до 44 лет, подписание информированного согласия, нормальная масса тела (ИМТ 18,5–24,9 кг/м²), отсутствие соматической патологии.

Критерии невключения: возраст младше 18 лет и старше 44 лет, хроническая соматическая патология, острые воспалительные заболевания в течение последнего месяца, применение про-, пре-, син-, мета-, антибиотиков, слабительных препаратов, а также препаратов, влияющих на моторику, за последние 3 месяца, вакцинация за последние 6 месяцев, травмы/оперативные вмешательства за последние 6 месяцев, злоупотребление алкоголем (потребление в неделю более 70 г этанола у женщин или 140 г этанола у мужчин), вегетарианство, беременность/лактация.

## Дизайн исследования

Проведено одноцентровое поперечное одномоментное контролируемое исследование.

## Методы

Все участники исследования заполняли анкету, специально разработанную под цели и задачи данного проекта. Всем участникам исследования проводилось антропометрическое обследование с измерением веса, роста, окружности талии (ОТ) и окружности бедер (ОБ), артериального давления (АД). Проводилась оценка ИМТ, который определялся как отношение массы тела в килограммах к квадрату роста в метрах (кг/м²).

Биохимическое обследование включало в себя определение общего холестерина (ОХ) холестеролоксидаза-пероксидазным методом (Mindray, Китай), липопротеидов высокой плотности (ЛПВП) (ммоль/л), липопротеидов низкой плотности (ЛПНП) (ммоль/л) и липопротеидов очень низкой плотности (ЛПОНП) (ммоль/л) прямым определением (Mindray, Китай), триглицеридов (ТГ) (ммоль/л) реакцией с глицерокиназой-пероксидазой (Mindray, Китай), мочевой кислоты (мкмоль/л) уриказно-пероксидазным методом (Mindray, Китай), уровня глюкозы (ммоль/л) ферментативным глюкозооксидазным методом (Mindray, Китай). Исследование биохимических показателей проводилось на биохимическом анализаторе BS-380 Mindray (Китай). Уровень гликированного гемоглобина (HbA1c, %) определяли измерением изменения флуоресценции запатентованного реагента, когда он вступал в реакцию с HbA1c в крови пациента, с использованием реагента EKF-diagnostic GmbH (Германия) на анализаторе Quo-Lab Analyser System (Германия). Уровень С-реактивного белка (СРБ) исследовали с помощью набора реагентов для высокочувствительного иммуноферментного определения концентрации CРБ в сыворотке крови, СРБ-ИФА-БЕСТ высокочувствительный. Диапазон измерений: 0–10 МЕ/л. Анализатор — абсорбционный микропланшетный ридер для 96-луночных планшетов Sunrise Bio-Rad TECAN (Австрия). Всем исследуемым проводился расчет индекса инсулинорезистентности HOMA-IR (HOmeostatic Model Assessment for Insulin Resistance) по следующей формуле:

HOMA-IR = Глюкоза [ммоль/л] х Инсулин [мкЕд/мл] ÷ 22,5.

Оценка состояния микробиоты толстой кишки проведена методом полимеразной цепной реакции в режиме реального времени (ПЦР-РВ) с применением набора реактивов «Колонофлор-16 (премиум)» (ООО «АльфаЛаб», Россия) с флуоресцентной детекцией результатов амплификации BioRad CFX96 (США). Набор «Колонофлор-16 (премиум)» объединяет наборы реагентов «Колонофлор-16 (биоценоз)» и «Колонофлор-16 (метаболизм), и позволяет количественно (lg KOE/г) определить общую бактериальную массу, Lactobacillus spp., Bifidobacterium spp., Bacteroides spp., Bacteroides thetaiotaomicron (B. thetaiotaomicron), Faecalibacterium prausnitzii (F. prau), Escherichia coli (E. coli), Akkermansia muciniphila (A. muciniphila), Enterococcus spp., E. coli enteropathogenic, Klebsiella pneumonia (K. pneumonia), Klebsiella oxytoca (K. oxytoca), Candida spp., Staphylococcus aureus (S. aureus), Clostridium difficile (C. difficile), Clostridium perfringens (C. perfringens), Proteus vulgaris/mirabilis (P. vulgaris/mirabilis), Citrobacter spp., Enterobacter spp., Fusobacterium nucleatum (F. nucleatum), Parvimonas micra (P. micra), Salmonella spp., Shigella spp., Blautia spp., Acinetobacter spp., Eubacterium rectale (E. rectale), Streptococcus spp., Roseburia inulinivorans (R. nulinivorans), Prevotella spp., Methanobrevibacter smithii (M. smithii), Methanosphaera stadmanae (M. stadmanae), Ruminococcus spp., соотношение Bacteroides spp./F. prau. Исследования выполнены на базе клинико-биохимической лаборатории Университетской многопрофильной клиники ФГБОУ ВО Тюменский ГМУ Минздрава России (зав. лабораторией — к.м.н. Южакова Н.Ю.).

## Статистический анализ

Для статистических расчетов был использован пакет прикладных программ Microsoft Exсel 2010, IBM SPSS Statistics 26.0. Качественные переменные представлены в виде абсолютных значений и частот (процентов). Количественные переменные описывались с помощью медианы и межквартильного интервала. Анализ взаимосвязей между двумя количественными переменными проводился с использованием корреляционного анализа с расчетом коэффициента корреляции Спирмена. Результаты оценивались как статистически значимые при уровне р<0,05.

## Этическая экспертиза

Протокол исследования был одобрен Этическим комитетом ФГБОУ ВО «Тюменский государственный медицинский университет» Министерства здравоохранения Российской Федерации на заседании от 13.03.23 г. № 113 (выписка из протокола заседания). Участниками было подписано информированное согласие.

## РЕЗУЛЬТАТЫ

## Объекты (участники) исследования

В исследовании приняли участие 118 молодых людей. Среди них — 87 пациентов с ожирением, и 31 человек входил в группу контроля. Группы статистически значимо не отличались по полу и возрасту (табл. 1).

**Table:** Таблица 1. Демографические и антропометрические характеристики пациентов с ожирением и контрольной группы

Параметр, Me [ Q1; Q3]	Контроль n=31	Ожирение n=87	p
Возраст, годы	29 [ 26,0; 34,0]	28 [ 23,0; 37,0]	0,542
Пол:			
мужчины, n (%)	15 (48,4%)	40 (46,0%)	1,000
женщины, n (%)	16 (51,6%)	47 (54,0%)	1,000
Вес (кг)	65,0 [ 58,5; 74,5]	109 [ 99,0; 125,5]	<0,001
ИМТ (кг/м²)	21,9 [ 20,2; 23,5]	37,2 [ 34,1; 42,05]	<0,001
Окружность талии (ОТ), см	76,0 [ 69,5; 83,0]	111,0 [ 104,0; 121,5]	<0,001
Окружность бедер (ОБ), см	98,0 [ 94,5; 100,5]	124,0 [ 117,0; 131,0]	<0,001
ОТ/ОБ	0,78 [ 0,73; 0,84]	0,92 [ 0,83; 0,97]	<0,001

Основную группу исследования составляли молодые пациенты с ожирением. Средняя продолжительность заболевания составляла 14 [ 9; 18] лет (табл. 2). 28 пациентов (32,9%) имели избыточную массу тела или ожирение с детского возраста, а 64 пациента (76,2%) признали наличие семейного анамнеза ожирения. Пиковый ИМТ в группе составлял 37,9 [ 35,9; 43,1] кг/м². Как в группе контроля, так и в группе ожирения, 87,1% участников родились естественным путем, а в 12,9% случаев путем кесарева сечения. Масса тела при рождении в группе пациентов с ожирением составляла 3500 [ 3145; 3800] г, в группе контроля 3400 [ 3170; 3535] г. На грудном вскармливании находились 83,3% пациентов с ожирением, средняя продолжительность которого составляла 9 [ 6; 14] месяцев. Необходимо сказать об особенностях пищевого поведения. 44,1% больных с ожирением отметили, что употребляют сладости ежедневно. 42,9% употребляют жирные (фастфуд) продукты до 3 раз в месяц. При этом только половина пациентов 54,7% принимают пищу 3–4 раза в день, а у 67,9% самый объемный прием пищи приходится на вечернее время. 90,6% человек хотя бы однократно в течение жизни предпринимали попытки снизить массу тела, из них 29,4% пациентов предпринимали более 10 попыток.

**Table:** Таблица 2. Общая характеристика клинико-анамнестических данных пациентов с ожирением и контрольной группы

	Контроль n=31	Ожирение n=87	p
Длительность избыточной массы тела или ожирения, годы Me [ Q1; Q3]	-	14 [ 9; 18]	
Наличие избыточной массы тела или ожирения в детском возрасте, n (%)	-	28 (32,9%)	
Пиковый ИМТ (кг/м²) Me [ Q1; Q3]	-	37,9 [ 35,9; 43,1]	
Наличие семейного анамнеза ожирения, n (%)	-	64 (76,2%)	
Способ родоразрешения: Рожденные путем кесарева сечения Рожденные естественным путем	4 (12,9%) 27 (87,1%)	11 (12,9%) 74 (87,1%)	1,000 1,000
Масса тела при рождении	3400 [ 3170; 3535]	3500 [ 3145; 3800]	0,479
Грудное вскармливание	26 (83,9%)	70 (83,3%)	1,000
Продолжительность грудного вскармливания, мес Me [ Q1; Q3]	6 [ 12; 18]	9 [ 6; 14]	0,352
Частота употребление сладостей (n, %) Не употребляют Употребляют редко (1–2 раза в год) Употребляют 1–2 раза в месяц Употребляют 1–2 раза в неделю Употребляют 2–5 раз в неделю Употребляют ежедневно	0 1 (3,2%) 0 5 (16,1%) 7 (22,6%) 18 (58,1%)	1 (1,2%) 1 (1,2%) 6 (7,1%) 28 (33,3%) 11 (13,1%) 37 (44,1%)	1,000 1,000 1,000 1,000
Частое употребление жирных продуктов (n, %) Не употребляют Употребляют редко (1–2 раза в год) Употребляют 1 раз в 2–3 месяца Употребляют 1–3 раза в месяц Употребляют 1–2 раза в неделю Употребляют 2–5 раз в неделю	8 (25,8%) 4 (12,9%) 4 (12,9%) 6 (19,4%) 9 (29,0%) 0	8 (9,5%) 10 (11,9%) 9 (10,7%) 36 (42,9%) 18 (21,4%) 3 (3,6%)	
Основной прием пищи приходится на вечернее время, (n, %)	11 (35,5%)	57 (67,9%)	0,002
Частота приемов пищи (n, %) 1–2 раза в день 2–3 раза в день 3–4 раз в день 4–5 раз в день Более 5 раз в день	4 (12,9%) 2 (6,5%) 23 (74,2%) 2 (6,4%) 0	12 (14,3%) 19 (22,6%) 46 (54,7%) 5 (6%) 2 (2,4%)	
Количество человек, предпринимавших попытки к снижению массы тела (n, %)	-	77 (90,6%)	
Не предпринимали попыток Предпринимали до 5 попыток Предпринимали от 5 до 10 попыток Предпринимали более 10 попыток		8 (9,4%) 36 (42,4%) 16 (18,8%) 25 (29,4%)
Курение в настоящий момент и в прошлом (n, %)	10 (32,3%)	49 (57%)	0,019

Методом ПЦР-РВ у всех пациентов с ожирением, в 100%, в составе кишечной микробиоты определялись симбиотические микроорганизмы — Bacteroides spp., Lactobacillus spp. и Bifidobacterium spp., а также E. coli и F. prau (табл. 3).

**Table:** Таблица 3. Сравнение частот обнаружения филотипов микроорганизмов в кале здоровых лиц и пациентов с ожирением

Филотипы, частоты обнаружения (абс., %)	Контроль n=29	Ожирение n=85	p
Lactobacillus spp.	29 (100%)	85 (100%)	-
Bifidobacterium spp.	29 (100%)	85 (100%)	-
Escherichia coli	29 (100%)	85 (100%)	-
Bacteroides spp.	29 (100%)	85 (100%)	-
Faecalibacterium Prausnitzii	29 (100%)	85 (100%)	-
Eubacterium rectale	29 (100%)	80 (94,1%)	0,327
Acinetobacter	28 (96,6%)	80 (94,1%)	0,612
Roseburia inulinivorans	27 (93,1%)	83 (97,6%)	0,267
Prevotella spp.	25 (86,2%)	74 (87,1%)	0,907
Bacteroides thetaomicron	23 (79,3%)	74 (87,1%)	0,367
Ruminococcus spp.	20 (69,0%)	67 (78,8%)	0,281
Streptococcus spp.	18 (62,1%)	57 (67,1%)	0,625
Blautia spp.	17 (58,6%)	45 (52,9%)	0,596
Enterobacter spp.	13 (44,8%)	48 (56,5%)	0,278
Staphylococcus aureus	10 (34,5%)	28 (32,9%)	0,879
Methanobrevibacter smithii	7 (24,1%)	20 (23,5%)	0,947
Methanosphaera stadmanae	5 (17,2%)	8 (9,4%)	0,311
Parvimonas micra	4 (13,8%)	20 (23,5%)	0,264
Clostridium perfringens	4 (13,8%)	16 (18,8%)	0,539
Proteus vulgaris/mirabilis	4 (13,8%)	7 (8,2%)	0,467
Fusobacterium nucleatum	3 (10,3%)	32 (37,6%)	0,005
Citobacter spp.	3 (10,3%)	5 (5,9%)	0,417
Escherichia coli enteropathogenic	2 (6,9%)	8 (9,4%)	0,679
Candida spp.	1 (3,4%)	6 (7,1%)	0,676
Enterococcus spp.	1 (3,4%)	4 (4,7%)	0,775
Klebsiella oxytoca	1 (3,4%)	2 (2,4%)	0,750
Clostridium difficile	1 (3,4%)	2 (2,4%)	0,750
Shigella spp.	1 (3,4%)	1 (1,2%)	0,446
Akkermansia muciniphila	0	5 (5,9%)	0,182

Один из основных продуцентов бутирата Eubacterium rectale встречается в 94,1% случаев пациентов группы ожирения, как и Acinetobacter spp. У 87,1% пациентов с ожирением выявлялись Bacteroides thetaomicron, один из метаболически наиболее активных видов бактероидов, и Prevotella spp., также принадлежащие к филуму Bacteroidetes. Roseburia inulinivorans и Ruminococcus spp., принадлежащие к филуму Firmicutes, были обнаружены у 97,6 и 78,8% пациентов с ожирением соответственно, а Akkermansia muciniphila (филум Verrucomicrobia) — у 5,9% пациентов с ожирением, при этом отсутствовала в группе контроля.

В составе микробиоты кишечника у пациентов определялись оппортунистические и патогенные виды микроорганизмов, среди которых чаще выявлялись гамма-протеобактерии: атипичные формы E.coli (Escherichia coli enteropathogenic 9,4%), Enterobacter spp. (56,5%), Candida spp. (7,1%). Кроме того, были выявлены другие условно-патогенные и патогенные бактерии: Proteus vulgaris/mirabilis (8,2%), Citobacter spp. (5,9%), Klebsiella oxytoca (2,4%), Staphylococcus aureus (32,9%). При определении видового состава микробиоты методом ПЦР-РВ также выявлены Clostridium perfringens (18,8%), Clostridium difficile (2,4%), Parvimonas micra (23,5%). В сравнении с группой контроля (10,3%), в группе ожирения достоверно превалирует Fusobacterium nucleatum (37,6%) (p=0,005). Также наблюдалось увеличение Prevotella spp., Blautia spp., Enterococcus spp. и Akkermansia Мuciniphila, что можно объяснить повышением в рационе углеводов, усилением их ферментирования, как следствие снижения рН среды и количества молочной кислоты. Следует отметить, что ни в группе контроля, ни в группе ожирения не были выявлены K. pneumonia (0%) и Salmonella spp. (0%), Shigella spp. имела минимальный процент 1,2%.

Во время анализа микробиоты выявлено, что общая бактериальная масса находится в пределах референсных значений. При анализе количественного распределения микроорганизмов отмечалась отчетливая тенденция к снижению содержания Lactobacillus spp. и Bifidobacterium spp. в группе ожирения (табл. 4).

**Table:** Таблица 4. Сравнение количественных показателей филотипов микроорганизмов в кале здоровых лиц и пациентов с ожирением Примечание: р — достоверность различий анализируемых показателей у контрольной группы и лиц с ожирением, * https://www.invitro.ru/analizes/for-doctors/535/65020/

Филотипы, количественные показатели Me [ Q1; Q3]	Норма «Колонофлор»*	Контроль n=29	Ожирение n=85	p
Общая бактериальная масса	1011–1013	1x1013 [ 4x1012; 3x1013]	1x1013 [ 3x1012; 4x1013]	0,982
Lactobacillus spp.	107–108	1x107 [ 5x106; 3x107]	6x106 [ 8x105; 3x107]	0,326
Bifidobacterium spp.	109–1010	3x1010 [ 1x109; 1x1011]	1x1010 [ 8x108; 7x1010]	0,217
Escherichia coli	106–108	2x108 [ 4x107; 7x108]	4x108 [ 6x107; 2x109]	0,156
Bacteroides spp.	109–1012	1x1013 [ 4x1012; 2x1013]	1x1013 [ 3x1012; 3x1013]	0,850
Faecalibacterium prausnitzii	108–1011	8x1011 [ 1x1011; 2x1012]	2x1011 [ 3x1010; 6x1011]	0,030
Отношение Bacteroides spp. и Faecalibacterium prausnitzii	0,01–100	33,33 [ 15; 62,5]	35 [ 12,5; 100]	0,498
Eubacterium rectale	108–1011	8x109 [ 4x108; 4x1010]	8x109 [ 5,5x108; 8x1010]	0,808
Acinetobacter spp.	≤106	8,5x106 [ 2x106; 3x107]	1x107 [ 3x106; 5,5x107]	0,317
Roseburia inulinivorans	108–1010	7x109 [ 1x108; 2x1010]	4x109 [ 2x108; 2x1010]	0,710
Prevotella spp.	≤1011	3x107 [ 4x106; 4x1010]	1x1010 [ 6x107; 4x1011]	0,029
Bacteroides thetaomicron	Любое	2x1010 [ 4x108; 4x1010]	4x109 [ 7x108; 2x1010]	0,553
Ruminococcus spp.	≤1011	2x108 [ 3,5x106; 1x109]	2x108 [ 1x107; 3x109]	0,446
Streptococcus spp.	≤108	6,5x106 [ 4x105; 4x107]	2x107 [ 3x106; 2x108]	0,086
Blautia spp.	108–1011	2x107 [ 2x106; 2x108]	1x108 [ 6x107; 3x109]	0,127
Enterobacter spp.	≤104	4x107 [ 4x105; 2x108]	1x107 [ 1x106; 1,5x108]	0,937
Staphylococcus aureus	≤104	2,5x106 [ 1x106; 1x107]	4,5x106 [ 1,5x106; 6x107]	0,482
Methanobrevibacter smithii	≤1010	6x106 [ 1x106; 1x107]	6x107 [ 6,5x106; 5x108]	0,063
Methanosphaera stadmanae	≤106	6x107 [ 2x107; 4x108]	8x107 [ 3,3x107; 9x107]	0,833
Parvimonas micra	0	2,5x108 [ 7,5x105; 4,5x1016]	3x106 [ 1x106; 5x108]	0,911
Clostridium perfringens	0	1,4x106 [ 5,5x105; 1,6x107]	3x106 [ 6x105; 8,5x106]	0,617
Proteus vulgaris/mirabilis	≤104	1,6x106 [ 2x105; 4,5x106]	8x105 [ 3x105; 4x106]	0,788
Fusobacterium nucleatum	0	2x105 [ 2x105; 7x106]	8x105 [ 4,5x105; 3,5x106]	0,379
Citobacter spp.	≤104	4x105 [ 2x105; 2x107]	3x106 [ 8x105; 1x107]	0,393
Escherichia coli enteropathogenic	≤104	1,2x104 [ 4x10³; 2x104]	1,35x106 [ 1,8 x104; 5,5x106]	0,636
Candida spp.	≤104	1x106 [ 1x106; 1x106]	2,5x106 [ 1x106; 2x1013]	
Enterococcus spp.	≤108	2x105 [ 2x105; 2x105 ]	1,5x1011 [ 4,51x107; 6,5x1011]	
Klebsiella oxytoca	≤104	0	0	
Clostridium difficile	0	3x1016 [ 3x1016; 3x1016]	1x1016 [ 4x107; 2x1016]	
Shigella spp.	0	7x105 [ 7x105; 7x105 ]	2x1016 [ 2x1016; 2x1016]	
Akkermansia muciniphila	≤1011	0	1x1010 [ 3x106; 4x1010]	

У пациентов с ожирением также наблюдалось достоверное снижение бактерий рода Faecalibacterium, в частности F. prau (p=0,030). Известно, что данная группа микроорганизмов относится к апатогенным, проявляющим защитные, регуляторные и пробиотические свойства, и обладает противовоспалительным потенциалом. Также данная группа бактерий участвует в синтезе КЦЖК и является главным продуцентом бутирата. Количество E. rectale, еще одного из микроорганизмов-лидеров по производству бутирата, находилось в пределах референсных значений.

В группе пациентов с ожирением было обнаружено значимо более высокое количество бактерий рода Prevotella spp. (p=0,029), относящихся к сапрофитным и условно патогенным, филотипам микроорганизмов, проявляющим протеолитические, гемолитические свойства, способность к продукции токсических факторов и обладающих провоспалительным эффектом. В отличие от группы контроля, в группе ожирения определялась A. muciniphila, относящаяся к филуму Verrucomicrobia, в наибольшей степени ассоциированному с метаболическими заболеваниями. Филотип A. muciniphila, не выявляющийся в группе контроля, относится к условно-патогенным бактериям, проявляющим протеолитические и гемолитические свойства, а также способность к продукции токсических факторов. Подтверждением патологических изменений микробиоты у лиц с ожирением также являлась отчетливо выраженная тенденция к увеличению количества F. nucleatum, относящегося к филуму Fusobacteria и обладающего провоспалительным потенциалом.

Кроме того, в группе ожирения отмечалась отчетливая тенденция к повышению патогенных микроорганизмов: Shigella spp. (2x1016 [ 2x1016; 2x1016]), C. perfringens (3x106 [ 6x105; 8,5x106]) и условно-патогенных микроорганизмов: Citobacter spp. (3x106 [ 8x105; 1x107]), E. coli enteropathogenic (1,35x106 [ 1,8x104; 5,5x106]), S. aureus (4,5x106 [ 1,5x106; 6 x107]).

Таким образом, опираясь на полученные данные, можно утверждать, что у молодых пациентов с ожирением по сравнению с аналогичными параметрами у здоровых лиц наблюдалось перераспределение филотипов микробиоты, характеризующееся уменьшением апатогенных микроорганизмов, появление и увеличение условно-патогенных и патогенных микроорганизмов, что в целом свидетельствует о формировании провоспалительного потенциала микробиоты при данной патологии.

Подтверждением данной гипотезы являются выявленные в ходе нашей работы многочисленные ассоциации представителей микробиоты толстой кишки с важнейшими антропометрическими, биохимическими параметрами у молодых пациентов с ожирением.

Так, обнаружены значимые отрицательные корреляционные зависимости общей бактериальной массы (r=-0,415, p=0,000), Lactobacillus spp. (r=-0,306, p=0,004), Bifidobacterium (r=-0,385, p=0,000), E. coli (r=-0,427, p=0,000), Bacteroides (r=-0,418, p=0,000), F. prau (r=-0,309, p=0,004), B. thetaomicron (r=-0,333, p=0,004), Acinetobacter (r=-0,413, p=0,000), E. rectale (r=-0,413, p=0,000), R. inuliniv (r=-0,240, p=0,029) и Ruminococcus (r=-0,343, p=0,005) с возрастом. Эти данные можно считать косвенным подтверждением гипотезы уменьшения разнообразия и активных свойств микробиоты с увеличением возраста. При этом условно-патогенный филотип A. muciniphila обнаруживает статистически значимую положительную корреляционную зависимость с возрастом (r=0,564, p=0,322).

С ИМТ и ОБ положительно коррелировали Bifidobacterium spp. (ИМТ r=0,275, p=0,011, ОБ r=0,400, p=0,000) и Acinetobacter spp. (ИМТ r=0,246, p=0,023, ОБ r=0,331, p=0,002). С ОБ положительно коррелировала общая бактериальная масса (r=0,239, p=0,027), Lactobacillus spp. (r=0,314, p=0,003) и C. perfringens (r=0,740, p=0,001). Со сроком рождения коррелировали Ruminococcus spp. (r=0,355, p=0,004), с продолжительностью грудного вскармливания — F. nucleatum (r=-0,493, p=0,009).

При анализе взаимосвязей параметров микробиоты и биохимических показателей было выявлено множество корреляционных зависимостей с параметрами липидного и углеводного обмена. Общая бактериальная масса имела отрицательную корреляционную связь с ОХ (r=-0,224, p=0,040). Bifidobacterium spp. отрицательно коррелировали не только с уровнем ОХ (r=-0,215, p=0,048), но и с уровнем ТГ (r=-0,276, p=0,011), ЛПНП (r=-0,235, p=0,030), ЛПОНП (r=-0,255, p=0,018), индексом АТГ (r=-0,247, p=0,023) и уровнем мочевой кислоты (r=-0,228, p=0,036).

F. prau и E. rectale отрицательно коррелировали с ТГ (r=-0,333, p=0,002 EP; r=-0,295, p=0,008 ER) и ЛПОНП (r=-0,337, p=0,002 ЕР; r=-0,291, p=0,009 ER). С ОХ и ЛПНП прослеживалась отрицательная корреляционная связь E. coli(ОХ r=-0,266, p=0,014; ЛПНП r=-0,311, p=0,004) и Bacteroides spp. (ОХ r=-0,247, p=0,023; ЛПНП r=-0,223, p=0,040).

У A. muciniphila (r=0,900, p=0,037) и E. сoli enteropathogenic (r=0,755, p=0,031) прослеживалась положительная корреляционная связь с уровнем ОХ. Candida отрицательно коррелировала с ТГ (r=-0,886, p=0,019), ЛПОНП (r=-0,886, p=0,019) и ЛПВП (r=-0,928, p=0,008). С ЛПВП прослеживалась положительная корреляционная связь у S. aureus (r=0,412, p=0,029). С уровнем ОХ (r=-0,244, p=0,024), ТГ (r=-0,237, p=0,029), ЛПОНП (r=-0,235, p=0,030) коррелировала Acinetobacter spp. С ЛПОНП также наблюдалась отрицательная корреляция R. inulinivorans (r=-0,233, p=0,034).

В отношении углеводного обмена была выявлена положительная корреляция с уровнем глюкозы у S. aureus (r=0,376, p=0,049), P. micra (r=0,461, p=0,041) и Blautia spp. (r=0,356, p=0,016). С уровнем инсулина и индексом НОМА-IR коррелировали Candida (инсулин r=-0,886, p=0,019; НОМА-IR r=-0,886, p=0,019) и M. stadmanae (инсулин r=-0,781, p=0,022; НОМА-IR r=-0,732, p=0,039). Наличие данных статистически значимых корреляционных зависимостей убедительно свидетельствует о существовании тесных и многообразных взаимосвязей количественных и качественных параметров микробиоты с метаболическими параметрами пациентов.

## ОБСУЖДЕНИЕ

Так как существуют трудно культивируемые бактерии, которые не выявляются бактериологическим методом, но играют существенную роль при ожирении, наш выбор остановился на использовании фекальной микробиоты методом ПЦР в реальном времени. В отличие от культурального, данный метод позволяет выделять ДНК даже тех бактерий, которые являются нежизнеспособными.

Результаты исследования состава микробиоты пациентов с ожирением в данном исследовании подтверждают наличие качественных и количественных изменений бактериально-грибковых ассоциаций кишечника. Изменения композиции филумов микробиоты толстой кишки могут являться триггером патологических метаболических нарушений, ассоциированных с системным воспалением при данной патологии [13]. Однако краеугольным камнем в интерпретации результатов параметров микробиоты при ожирении является вопрос «нормы». Сравнивая количественные показатели микробиоты толстой кишки обследуемых с формально-нормативными, приведенными в стандарте «Колонофлор премиум» и результатами пациентов группы контроля, мы видим отличия, характеризующиеся снижением и повышением некоторых филотипов (F. prau, Bifidobacterium spp., Bacteroides spp., Blautia spp., Enterobacter spp. и др.). Результаты исследований, в частности проекта Human Microbiome Project (HMP) [14], основанные на индикации 16SpРНК бактерий, убедительно доказали, что понятие «норма» зависит от множества факторов: возраста, этнической принадлежности, приема препаратов, различных групп в анализе и других факторов [14], что позволяет нам, основываясь на результатах данного исследования, сформировать предложения по новым референсным значениям.

Различия в составе микробиоты кишечника между пациентами с ожирением и лицами с нормальной массой тела начинаются на уровне рода. В группе пациентов с ожирением наблюдается тенденция к увеличению бактерий рода Blautia и Enterococcus, при этом достоверно преобладает род грамотрицательных анаэробных неспороносных палочкообразных бактерий Prevotella spp. (Bacteroides) (р=0,029). Имеются сообщения о связи Prevotella spp. с риском развития метаболического синдрома [15]. Нужно отметить, что бактерии рода Prevotella обладают провоспалительными свойствами, реализующимися через способность стимулировать выработку провоспалительных цитокинов. В нашем исследовании выявлена ассоциация бактерий рода Prevotella с СРБ (r=0,285, p=0,014).

Провоспалительные свойства характерны не только для бактерий рода Prevotella, но и для F. nucleatum (Fusobacteria), статистически достоверно превалирующей в группе лиц с ожирением (р=0,005). Провоспалительное микроокружение, создаваемое F. nucleatum за счет токсинов FadA, Fap2 и ЛПС, благоприятствует развитию неопластических процессов в толстой кишке, а также развитию и прогрессированию системных заболеваний [16]. С другой стороны, Fusobacterium производит значительное количество масляной кислоты, которая является основным источником энергии для колоноцитов. В нашем исследовании была выявлена отрицательная ассоциация F. nucleatum с длительностью грудного вскармливания (r=-0,337, p=0,002).

Еще одним важным микроорганизмом, продуцирующим бутират и занимающим 5–15% состава микробиоты кишечника человека, является F. prau. Уровень данной комменсальной грамположительной анаэробной палочки достоверно ниже в группе ожирения (p=0,030). Это согласуется с результатами ряда исследований, которые работали с пациентами с ожирением и диабетом, пациентами с ожирением, метаболическим синдромом и НАЖБП [17][18]. Однако есть противоречивые данные, например, раннее исследование, проведенное на юге Индии, выявившие значительно более высокую численность F. prau у детей, страдающих ожирением, по сравнению с детьми, не страдающими ожирением [19].

В многочисленных исследованиях описаны противовоспалительные свойства F. Prau. Снижение F. prau связано с развитием хронического воспаления, что наблюдается при ожирении. В многочисленных исследованиях описаны противовоспалительные свойства F. prau, способность улучшать целостность кишечного барьера за счет стимуляции секреции муцина, уменьшать бактериальную транслокацию, которые, по-видимому, связаны с его способностью производить бутират. Снижение F. prau связано с развитием хронического воспаления, что наблюдается при ожирении

Таким образом, использование F. prau или продуктов его производных может представлять собой хорошую альтернативу для лечения кишечных расстройств, связанных с ожирением и сопутствующими заболеваниями. Кроме того, мы можем идентифицировать виды, которые менее распространены при ожирении и связанных с ним расстройствах, что может быть связано с положительным воздействием на ожирение.

## ВЫВОДЫ

В ходе исследования изучены особенности микробного состава толстой кишки у молодых пациентов с ожирением и группы здоровых лиц. Выявлено, что результаты группы контроля расходятся с нормативными параметрами, приведенными в стандарте «Колонофлор-16 премиум», что, возможно, позволит нам сформировать новые референсные значения параметров микробиоты толстой кишки в будущем.

Наблюдается перераспределение филотипов микробиоты, характеризующееся уменьшением апатогенных микроорганизмов и появлением и увеличением условно-патогенных и патогенных микроорганизмов, что в целом свидетельствует о формировании провоспалительного потенциала доминантов и ассоциантов у молодых пациентов с ожирением.

Кроме того, в ходе исследования установлено множество ассоциаций микробных представителей с важнейшими антропометрическими, анамнестическими, биохимическими и метаболическими параметрами.

Установленные закономерности имеют принципиальное значение для понимания механизмов формирования патогенетических нарушений, приводящих к ожирению. Безусловно, стоит продолжать исследовательскую работу в данном направлении, однако уже сейчас становится очевидным, что воздействие на микробиоту толстого кишечника и ее метаболиты, в частности добавление F. Prau, в будущем может стать многообещающим подходом к терапевтическим вмешательствам и лечению ожирения и связанных с ним метаболических заболеваний.

## ДОПОЛНИТЕЛЬНАЯ ИНФОРМАЦИЯ

Конфликт интересов. Авторы декларируют отсутствие явных и потенциальных конфликтов интересов, связанных с содержанием настоящей статьи.

Источники финансирования. Работа выполнена по инициативе авторов без привлечения финансирования.

Вклад авторов. Все авторы одобрили финальную версию статьи перед публикацией, выразили согласие нести ответственность за все аспекты работы, подразумевающую надлежащее изучение и решение вопросов, связанных с точностью или добросовестностью любой части работы.
